# Moyamoya angiopathy: long-term follow-up study in a Finnish population

**DOI:** 10.1007/s00415-018-9154-7

**Published:** 2018-12-17

**Authors:** Marika Savolainen, Satu Mustanoja, Johanna Pekkola, Tiina Tyni, Anna-Maria Uusitalo, Sanni Ruotsalainen, Erja Poutiainen, Juha Hernesniemi, Leena Kivipelto, Turgut Tatlisumak

**Affiliations:** 1 Department of Neurology, South Karelian Central Hospital, Valto Käkelän katu 1, 53130 Lappeenranta, Finland; 2 Department of Neurology and Clinical Neurosciences, University of Helsinki and Helsinki University Hospital, Helsinki, Finland; 3 Department of Radiology, HUS Medical Imaging Center, Helsinki University Hospital and University of Helsinki, Helsinki, Finland; 4 Department of Child Neurology, Helsinki University Hospital and University of Helsinki, Helsinki, Finland; 5 Institute for Molecular Medicine Finland FIMM, University of Helsinki, Helsinki, Finland; 6 Department of Clinical Neurosciences, University of Helsinki, Helsinki, Finland; 7 Juha Hernesniemi International Center for Neurosurgery, Henan Provincial People’s Hospital, Zhengzhou, China; 8 Department of Neurosurgery and Clinical Neurosciences, University of Helsinki and Helsinki University Hospital, Helsinki, Finland; 9 Department of Clinical Neurosciences, Institute of Neuroscience and Physiology, Sahlgrenska Academy at University of Gothenburg, Gothenburg, Sweden; 10 Department of Neurology, Sahlgrenska University Hospital, Gothenburg, Sweden

**Keywords:** Stroke, Moyamoya angiopathy, Treatment, Prognosis, Follow-up, Quality of life, Mood symptoms

## Abstract

**Background and purpose:**

Moyamoya angiopathy (MMA) is a chronic cerebrovascular disorder predominantly starting in childhood or early adulthood and thus affects the whole lifetime. Little is known on MMAs long-term outcomes in European patients. We report long-term follow-up data on Finnish MMA patients.

**Methods:**

We included patients from our Helsinki University Hospital MMA database and arranged long-term follow-up visits for all the patients. This follow-up included a review of the medical records accumulated in due time, detailed neurological and neuropsychological evaluation, and outcome measures modified Rankin Scale (mRS) and Barthel Index (BI).

**Results:**

There were 61 MMA patients with a mean follow-up period of 9.5 years (SD 6.7 years; range 1.3–35.4 years; 581 patient-years). Only two patients had died and two-thirds (*n* = 40, 65.6%) had no new events during the follow-up period. Eight patients (13.1%) had an ischemic and five patients (8.2%) a hemorrhagic stroke during the follow-up. There were no differences between operated (*n* = 26) and conservatively (*n* = 35) treated groups regarding recurrent events or the outcome measured with mRS or BI. Finnish MMA patients reported significantly poorer physical and psychological health aspects of QOL when compared to the general Finnish population. Symptoms of low mood were found in 27 (56%) patients.

**Conclusions:**

Finnish MMA patients have a benign and stable course with a ~3.5 % annual stroke risk. We found no differences in the clinical outcomes between the operated and conservative groups, however, the psychosocial well-being requires more attention in MMA patients.

## Introduction

Moyamoya angiopathy (MMA) is a rare progressive steno-occlusive cerebrovascular disorder mostly affecting the anterior circulation, and more specifically the distal portion of the internal carotid artery and its distal branches, and only occasionally the posterior circulation. Insidious occlusion of the anterior circulation cerebral arteries leads to formation of the characteristic abnormal vascular networks, i.e. the moyamoya vessels [1]. MMA is a chronic disorder starting in childhood or early adulthood with a clear dominance in females, leading to a life-long affection. Although MMA is less frequent in Europe, accumulating evidence suggests it to be substantially more common than previously believed. Germans have done studies in their patient population concerning recurrent stroke risk in surgically and conservatively treated patients [2], and efficacy of STA–MCA bypass surgery in Caucasian patients [3]. Despite this, the natural course of the disease, i.e. its long-term events and outcomes, as well as patients’ quality of life and state of mood, are not thoroughly studied in the other European populations.

After establishing the Helsinki University Hospital-Moyamoya Angiopathy (HUH-MMA) database including 61 Finnish origin MMA patients [4], we arranged a prospective follow-up study of our patients. Our aim was to find out the long-term consequences and prognostic characteristics of the Finnish MMA population including the vocational outcomes considering the young age of most of the patients, and self-reported quality of life (QOL) and state of mood.

## Materials and methods

This study was performed at the Departments of Neurology and Neurosurgery, in Helsinki University Hospital (HUH), Helsinki, Finland. The local ethics committee approved the study (154/13/03/00/10). All MMA patients who agreed to come for an outpatient clinical follow-up visit gave a written consent. For registry-based data collection, Finnish legislation does not require patient consent.

We searched for all diagnosed moyamoya disease (MMD) and moyamoya syndrome (MMS) patients referred to our hospital and collected the patient data into a detailed database as reported earlier [4] and called them for a face-to-face follow-up visit. Our HUH-MMA database includes detailed data on the patient’ medical history, family history of stroke and MMA, medication on admission and preventive medication at discharge, hospital admission data, clinical manifestation and time course, treatment and procedures, discharge details, laboratory tests on admission, radiological data, all deaths, vascular deaths, and causes of deaths in detail.

This was a cross-sectional clinical follow-up study. The follow-up data of the deceased patients and of those who wished not to participate in the study were followed-up from the death certificates and hospital records. All consenting patients were personally interviewed by an experienced neurologist (MS). The follow-up period started after the patient had visited the hospital for the first time due to the MMA-related symptoms and ended at the end of September 2015 when the hospital charts were last checked.

The clinical examination at the follow-up visit included the National Institutes of Health Stroke Scale (NIHSS) score, modified Rankin Scale (mRS), and Barthel Index (BI). The working status (employed/working/studying, permanent disability pension, retired due to age, sick leave) was recorded. A new stroke, any other new diagnosis after the MMA diagnosis, the medication, surgical operations, perioperational complications, and family history of strokes or MMA were recorded, based on the interview and the medical records.

We defined progression of the disease as a new ischemic or hemorrhagic stroke after the primary hospital admission or any worsening in a stenotic vessel according to visual examination of the angiography images and development of stenosis in a vessel that was normal-looking in the previous images. Favorable outcome was defined as mRS 0–2 and an excellent outcome as mRS 0–1.

Questionnaires concerning the quality of life and the profile of mood were sent to the patients by mail. The patients returned the filled questionnaires at the beginning of the comprehensive neuropsychological examination. The questionnaires were checked after the enquiry and completed together with the patient if there were missing items. A psychologist (AMU), familiar with the neuropsychological assessment procedure, conducted all neuropsychological examinations and she was blinded to the current clinical neurological and neuroradiological data.

### Quality of life

WHOQOL-BREF has been used worldwide in diverse diseases and conditions to measure the quality of life [5]. WHOQOL-BREF includes 26 items and four domains: physical health, psychological health, social relationships, and environment [6]. The items are computed to four separate domains and separately scaled from 0 to 100, where 0 is the worst possible score and 100 the best. The questionnaire also includes questions of perceived health in general (how satisfied are you with your health?) and quality of life in general (how would you rate your quality of life?). The questionnaire has a 5-point Likert-scale (1 = very poor to 5 = very good). Perceived quality of life in MMA patients was compared to a Finnish population-based sample survey [7].

### Mood state

Modified profile of mood states (POMS) questionnaire was used to assess patients’ mood symptoms [8, 9]. We used a total score of the modified version of the POMS that includes 38 adjectives rated on a 5-point Likert scale (0 = not at all to 4 = very much). Patients were asked to circle adjectives which describe feelings and emotions during last week [9]. The characteristics of the controls of a previous Finnish study [10] were used to determine a cut-off point for elevated mood in our MMA patients. The elevated mood was determined as POMS score higher than 41.4 (i.e. 75 percentile of the control group, unpublished data).

### Statistical analyses

Continuous data are reported as mean ± SD. All statistical analyses were done using the SPSS software (IBM Corp. Released 2012. IBM SPSS Statistics for Windows, Version 22.0. Armonk, NY: IBM Corp.). We compared two groups: those treated with a revascularization by-pass operation to those treated conservatively using Crosstabs function and Kaplan–Meier. Actual annual stroke risk with 95% confidence intervals (CI) was calculated with the Life tables function using the formula 1 − [(1 − Ic)^1/*n*^], with Ic as cumulative incidence rate and n as the number of years. Quality of life and mood symptoms were compared between by-pass operated and conservatively treated patient groups using an independent sample *T* test with 95% confidence. One sample *T* test was used to compare MMA patients QOL with a Finnish population-based sample survey [7]. A two-sided probability (*P*) value of 0.05 or lower was considered significant.

## Results

All 61 patients were followed-up. Two patients died during the follow-up period and four patients either did not want to participate or could not be reached. Their death certificates and medical records were analyzed. Fifty-five patients came to the follow-up visit. The data were obtained by interviewing the patients and their relatives, as well as by going through the medical records. The mean age at the start of the follow-up was 35 (SD 17; range 3–77). Most patients (*n* = 50, 82%) were women. The mean follow-up period was 9.5 years (SD 6.7 years; range 1.3–35.4 years). Patient-years summed up to 581.

*Mortality* Two female patients died during the follow-up period. Neither of them was using antiplatelet therapy. One of them was diagnosed with MMD at the age of 50 years after an intraventricular hemorrhage. A year later she underwent bilateral revascularization surgery (STA-MCA + EDAMS). She died 4 years later after a new intracerebral hemorrhage (ICH). The second patient was diagnosed at the age of 19 after a transient ischemic attack. On digital subtraction angiography bilateral internal carotid artery occlusion and moyamoya collateral network were detected. At the age of 47 and 48 she had vertigo and balance problems and imaging showed microhemorrhages as well as typical bilateral MMA vessel abnormalities. She died at the age of 53 because of ICH.

*New vascular events* Two-thirds (*n* = 40, 65.6%) of the patients had no new vascular events. Eight patients (13.1%) had an ischemic and five patients (8.2%) had a hemorrhagic stroke during the follow-up. The average annual rate of a recurrent stroke from the first event for all study subjects was 3.5%. There were no differences between the operated and the conservatively treated patients in the stroke recurrence risk. In seven patients (11.5%) we detected asymptomatic progression in their vascular stenosis. During the follow-up period, one of the unilateral MMS patients progressed to bilateral MMS. Three out of 8 (37.5%) patients initially presenting with a hemorrhagic stroke had a new hemorrhagic event during the follow-up, and 6 out of 31 patients (19.4%) initially presenting with an ischemic stroke had a new ischemic stroke during the follow-up (Table 1).

**Table 1:** Presenting data on MMD patients with new vascular events during the follow-up period

Sex, birth year	MMD classification	Presenting symptom	Year	Time to event (months)	Follow up symptom/event	Year	mRS on	Type of surgical treatment and year	Events after surgical treatment
M 1959	MMD definitive	IVH	1998	113	SAH	2007	1		
M 1975	MMD unilateral	CI	1983	26	CI	2009	0		
M 1957	MMD definitive	Seizure	2006		Progression of stenosis		0		
F 1986	MMD definitive	CI	2013		Progression of stenosis		2		
F 1988	MMS (Down)	Stenosis l.sin			Stenosis l. dx			EDAS l. sin. 2006	–
F 1958	MMD definitive	ICH	2003	101	ICH	2011	2	STA–MCA bilateral 2004	ICH
F 1960	MMD definitive	CI	2013	7	CI	2014	0	**	–
F 1965	MMD definitive	Tinnitus	2005		Old bleed in MRI, asymptomatic*		0		
F 1965	MMD definitive	CI	2004		CI × 2	2005, 2009	1		
F 1959	MMD definitive	CI	1983	268	CI (dg)	2005	2		
F 1985	MMD definitive	CI	2008		Progression in stenosis	2013	0	**	
F 1959	MMD definitive	IVH	2009	50	ICH	2014	6 (2014)	STA–MCA + EDAMS bilateral 2010	ICH 2014
F 1959	MMD definitive	TIA	1978	431	ICH	2013	6 (2013)		
F 1971	MMD definitive	CI	1995	99	CI	2003	1		
F 1966	MMD definitive	CI	2006	33	CI	2009	2	STA–MCA 2006, 2007	CI 2009
F 1996	MMD definitive	CI	2005		Progression in stenosis on left	2006	0	EDAS l. dx. 2005, l. sin 2006	–
F 1986	MMD definitive	CI	2006	12	Progression in stenosis on left	2008	1	STA–MCA l. dx. 2006, l. sin. 2007	Progression of stenosis without symptoms
F 2001	MMD definitive	CI	2009		Post. op. TIA		0	EDAMS 2009 l. dx	TIA
F 1969	MMD definitive	SAH	1998		Progression of stenosis	2005	1		
F 1970	MMD definitive	CI	2013	15			1	STA–MCA l. dx. 2013, l.sin. 2014	–
F 1983	MMD unilateral	Seizure	2002		Progression of stenosis	2006	0	ECA–MCA l.dx. 2003	–

*CI* Cerebral infarction, *ECA–MCA* external carotid artery–middle cerebral artery bypass, *EDAS* encephalo-duro-arterio-synangiosis, *EDAMS* encephalo-duro-arterio-myo-synangiosis, *F* female, *ICH* intracerebral hemorrhage, *IVH* intraventricular hemorrhage, *M* male, *MMD* moyamoya disease, *MMS* moyamoya syndrome, *mRS* modified Rankin Scale, *SAH* subarachnoid hemorrhage, *STA–MCA* superficial temporal artery–middle cerebral artery bypass, *TIA* transient ischemic attack

*Old bleed appeared on follow-up imaging, but has been asymptomatic

**Operated after our follow up visit

*Neurological outcomes* The mean NIHSS at follow-up was 1 and median 0. The mean/median mRS at follow-up was one. The follow-up examination was done 0.2–11.7 years after recurrent strokes, in 12 patients. Functional outcome was favorable (80%) or excellent (71%) in most of the patients. There was no difference in the outcome between the operated (*n* = 26) and conservatively treated (*n* = 35) patients, when measured with mRS or BI. The mean BI at follow-up was 97 (SD 12) and median 100 (min 25). Only 10% of the patients required assistance in activities of daily living.

*Surgical treatments* Twenty-six patients underwent revascularization surgery. The techniques used are reported in detail previously [4]. One patient had an intraoperative complication, a small arterial wall laceration. There was one postoperative hematoma which was treated conservatively. However, this patient was later shunted for communicating hydrocephalus. Three patients had wound healing problems.

*Medical treatments* 70% of the conservatively treated patients used antiplatelet or anticoagulation (*n* = 1) therapy and 69% of the operated patients used antiplatelet therapy at the follow-up visit [4].

*Vocational outcomes and psychosocial factors* More than half (59%) of the patients were working, while 32% were on a permanent disability pension due to MMA, 5% had retired due to age, and 3% were on sick leave at the follow-up visit.

*Quality of life* The WHOQOL-BREF questionnaire was available for 48 MVV patients. The patients reported significantly poorer physical health and psychological health aspects of QOL and a trend for poorer environmental aspects of the QOL when compared to the Finnish population-based sample survey (Table 2).

**Table 2:** Differences in quality of life scores between the patients with MMA and Finnish population-based sample [7]

WHOQOL-BREF	MMA patients *N* = 48, mean (SD)	Sample survey *N* = 4306, mean (SD)	*P* value*
Physical domain	66.7 (21.9)	77 (17.4)	0.002
Psychological domain	64.9 (18.9)	74 (14.4)	0.002
Social relationship	74.6 (19.0)	79.0 (16.8)	0.119
Environmental domain	72.2 (17.5)	77 (12.9)	0.067

WHOQOL-Bref = Quality of life scales [6]

*One sample *T* test

The surgically treated patients reported better social relationship aspects of QOL compared to conservatively treated patients (mean ± SD 81.0 ± 17.2 vs. 69.7 ± 19.2; *P* = 0.04). There were no significant differences in the other three subdomains (physical, psychological, and environmental domains) of QOL, however, the working MMA patients estimated their physical, psychological, and social aspects of QOL to be better than patients on sick leave or pension (Table 3).

**Table 3:** Differences in quality of life and mood symptoms between the working MVV patients and patients at sick leave or pension

Psychosocial functioning	Currently at work life *N* = 29, mean (SD)	Sick-leave or pension *N* = 19, mean (SD)	*P* value*
WHOQOL-BREF			
Physical domain	75.3 (17.7)	53.5 (21.3)	0.000
Psychological domain	71.0 (15.9)	55.7 (19.7)	0.005
Social relationship	79.4 (15.0)	67.4 (22.4)	0.032
Environmental domain	76.2 (16.6)	66.2 (17.5)	0.288
POMS [6, 7]	42.3 (26.9)	50.0 (20.1)	0.295

POMS = Profile of Mood States [8]

WHOQOL-Bref = Quality of Life Scales [6]

*Independent samples *T* test

*Mood state* POMS was obtained from 48 MMA patients. Mean POMS total score was 45.4 ± 24.5. Symptoms of low mood were found in 27 (56%) patients, using a cut-off point of 41.4 points derived from the scores of the previous Finnish study [10]. Surgically- and conservatively-treated patients did not differ significantly in POMS total score (39.3 ± 25.4 vs. 50.1 ± 23.2; *P* = 0.13). Working status (i.e. work, sick leave, and pension) was not associated with patients’ mood symptoms.

## Discussion

We prospectively collected detailed follow-up and outcome information on all the 61 Finnish MMA patients diagnosed and included in our registry. During a total of 581 patient-years, two patients had died (due to ICH), 13.1% (*n* = 8) had an ischemic and 8.2% (*n* = 5) a hemorrhagic stroke, and 42.6% (*n* = 26) had undergone revascularization surgery, while two-thirds (65.6%) had no new vascular events. Functional outcome was favorable in 80% and excellent in 71% of the patients. 55% of the patients were working at the time of the follow-up visit. There was no difference between patients with surgical treatment vs. those treated conservatively considering the NIHSS, mRS, BI, mood symptoms, or working status at the follow-up visit. MMA patients showed poorer QOL measures compared to healthy control subjects.

There are not many systematic, long-term follow-up studies of MMA patients, making it difficult to reliably inform the patient and their families about prognosis, develop prognostic scores, estimate the best preventive approaches, or suggest more aggressive treatments in patients who are expected to have a poor outcome. Furthermore, the characteristics of MMA disease seem to be different in various patient populations with different ethnical backgrounds [11]. The major studies reporting long-term outcomes of MMA patients have included 1146 [12], 104 [13], and 101 [14] patients with 5.2–9 years, 29–46 months, and 26.5 months of follow-up time reported.

In a small German study the mortality rate was 9.5% (2 patients) during a mean follow-up period of 3.7 years [2]. In a study from the United States the overall mortality rate after surgical revascularization was 2.3% during a mean follow-up period of 4.9 years in 233 adult and 96 child MMA patients [15]. In a Korean study with a mean follow-up of 82.5 months the mortality rate was 6.5% and the cause of death in all four cases was recurrent hemorrhage [16]. Our 3% mortality during a 9.5-year period (mean follow-up) is partly in line with these, or even less than that in the previous studies. Interestingly, ICH appears to be the main cause of death in all published series.

In the Korean population, the reported annual risk of stroke was 4.5%/person-year, with 5- and 10-year cumulative risks of any stroke being 17% and 30%, respectively [16]. Furthermore, patients presenting with a hemorrhagic event tended to show a higher incidence for a recurrent hemorrhage and patients with ischemic symptoms had a higher rate of recurrent ischemia [16] which is in line with our results. One Japanese study reported the recurrence rate for cerebral infarction to be lower in the surgery group than the non-surgery group, although the rate of infarction was not different between the antiplatelet and the non-antiplatelet subgroup [12]. They also found no difference in the recurrence rate of cerebral hemorrhage in patients whose initial symptom was hemorrhage when treated surgically or conservatively [12]. In a Chinese study, the investigators found the annual rebleeding rate to be 2.2% following encephaloduroarteriosynangiosis operation in 95 MMV patients during a median follow-up of 8.5 years [17]. Another Korean study found that 86% of adult MMD with conservative management did not develop recurrent ischemic stroke after an ischemic stroke when 90% of the patients used antiplatelet medication [13]. Our results are in line with this Korean study. In a German MMA patient population (*n* = 21) the 5-year-Kaplan–Meier risk for recurrent stroke was 80.95%. 11 patients (52.3%) had the neurosurgical revascularizing procedure done and the Kaplan–Meier risk of perioperative or subsequent stroke was 27.27% within the first month and was stable thereafter. They found no significant difference in the number of strokes between surgically and medically treated patients [2].

In the German study, 68.4% of 21 patients did not have significant long-term disability (mRS 0 or 1) [2]. In the American revascularization study the mean mRS was 0.83 at the mean follow-up of 4.9 years [15]. In the Korean study mRS was 1.3 ± 1.4 after 82.5 months of follow-up [16]. Our results are in good line with all of these existing reports. In general, MMA appears to affect the patients mildly both in Asian and Western populations including our Finnish patient population.

Extracranial–intracranial by-pass operation is usually considered to be the treatment of choice for MMA patients presenting with ischemic stroke, despite a lack of firm evidence [12, 18]. For hemorrhagic stroke the evidence is even less clear, although the JAM trial suggested a statistically marginal preventive effect of direct bypass against rebleeding [19], especially for patients with posterior circulation region hemorrhages [20]. In a recent meta-analysis based on Asian patient series, surgical revascularization was superior to conservative treatment in reducing the rate of recurrent stroke events including both ischemic and hemorrhagic events but not in reducing mortality [21]. Another recently published meta-analysis mainly including articles concerning studies done in Asian populations, also two articles including two Caucasian MMA patient populations, found that surgery significantly decreased the future stroke events compared to conservative treatments in adult MMD [22]. A third meta-analysis including mainly studies in Asian, but also five Caucasian populations, found that the surgical treatment significantly reduced the risk of stroke and according to subgroup analysis the surgical treatment was more beneficial in hemorrhagic MMD [23]. In a German study of 54 Caucasian MMA patients, where all patients underwent STA–MCA bypass operation and were followed-up for 38.2 months thereafter, nearly all patients remained symptom-free from ischemic strokes, hemorrhages, and microbleeds. The authors, therefore, considered bypass surgery safe and effective in Caucasian MMA patients [3]. Antiplatelet therapy is commonly used for patients initially presenting with ischemic symptoms [24] despite lack of evidence for its efficacy [12]. It is often difficult to predict the clinical course of a single patient, and therefore, makes it difficult to recommend either operation or antiplatelet treatment without firm evidence.

Among our patients, those who did not receive by-pass operation were either severely handicapped due to a stroke, or were nearly symptom-free, and therefore, not willing to take a potential risk of surgery. One-third of our patients had not been referred to neurosurgical consultation at all, as some physicians have doubts on efficacy and safety of surgical treatment because of the lack of published randomized studies. These various factors create a significant selection bias making the surgery and conservative treatment groups even less comparable. While surgery is often offered after clinical judgement the evidence for surgery is fairly weak. In the future, however, larger multicenter datasets will help to compare conservative and surgery-treated patients with baseline characteristics being adjusted with a propensity-score approach and deliver better-quality data for designing randomized trials.

Vocational outcomes in MMA patients have not been published earlier. Our study shows that despite our patients being fairly young, only 59% could return to work, and even one-third (32%) became disability-pensioned despite good mRS and BI scores. These findings require precise evaluation for the exact cause of disability to seek possible interventions and suitable employment strategies for the young MMA patients. Cognitive decline occurring over several years often attributed to chronic hypoperfusion is often suggested in MMA patients, but has not yet been adequately investigated.

Our MMA patients estimated their QOL less satisfying in physical and psychological domains when comparing to the Finnish population-based sample [7]. This finding is in line with previous results reporting that chronic diseases (i.e. stroke or other neurological diseases) are known to diminish the QOL [5, 25]. It has been suggested that the awareness of MMD may develop severe disturbances in performance and could be the main reason for psychological symptoms caused by MMD [26]. QOL was perceived lower among those MMA patients who were currently not working when compared to those who were in work life. Our finding is in line with a study where better QOL and fewer signs of low mood were associated with return to work after a mild stroke [27].

So far there is no curative treatment to MMD itself and the uncertainty of the progress of the disease may raise doubts about patients’ well-being and health in the future. Long-term depressive symptoms [28, 29] as well as diminished QOL [28–30] are frequently found among stroke patients. In this study, symptoms of low mood were found in slightly over half (56%) of Finnish MMA patients. However, the working status was not significantly related to a perceived state of mood among MMA patients. In previous studies MMA patients’ reported mood symptoms have been slightly lower compared to our study. Almost one-third of MMD patients in the Karzmark et al. study expressed symptoms of low mood, namely depression (28%) and anxiety (29%) [31]. In the study of Festa et al. 28% of the MMD patients developed moderate or severe depression [32].The slight differences in the frequencies of mood symptoms between these studies and our study may be explained by the differences in methodology. The mood scale in our study taps general symptoms, whereas in earlier studies more diagnostic measures have been used.

MMD itself may be responsible for depression and reduced QOL [26]. Caucasian patients with MMD are at a considerably higher risk of future strokes, even though the risk appears to abate over time [33]. These issues may have an impact to perceived QOL, as well as experienced state of mood in MMA patients. Psychological factors in chronic diseases like MMA affect the general well-being and may complicate the medical care. QOL is important to assess already during the diagnostic process and especially when treatment and rehabilitation are planned. So far, the impact of psychosocial factors has not been fully understood among MMA patients, although it seems that these factors are important for subjective well-being and have an impact even on the work status. In the future, we need larger, well-designed longitudinal studies to fully understand the consequences of MMA in patients from various geographies to gain better insight into the disease and hopefully its treatments.

Our study has several merits. First, we could include all diagnosed patients within our catchment area, closely approaching to the population-based setting. We investigated the patients in detail, both retrospectively and prospectively. Almost all patients were personally met and examined by a neurologist. We had a rather long follow-up time. Furthermore, the patient population is homogeneous all being of Finnish origin and living in Finland. However, we are also aware of some limitations. The number of patients is modestly reflecting the rarity of MMA in European populations.

Our study indicates a relatively benign course of MMA in the long run in Finnish patients, but only slightly over half of the patients were actively working. Multicenter prospective long-term follow-up studies and randomized trials with substantially larger patient numbers are needed in the future to obtain more vigorous data and to test the benefits of various treatment approaches.
